# Methylated septin 9 gene is an important prognostic marker in stage II and stage III colorectal cancer for evaluating local recurrence or distant metastasis after surgery

**DOI:** 10.1186/s12876-022-02172-6

**Published:** 2022-02-28

**Authors:** Mingliang Huang, Jiehua He, Wei Lai, Lu Liu, Heyang Xu, Yujie Zeng, Qiusheng Lan, Xiangan Lin, Zhonghua Chu

**Affiliations:** 1 Guangdong Provincial Key Laboratory of Malignant Tumor Epigenetics and Gene Regulation, Department of Gastrointestinal Surgery, Sun Yat-Sen Memorial Hospital, Sun Yat-Sen University, 107 Yan Jiang West Road, Guangzhou, 510120 China; 2 Department of Oncology, Sun Yat-Sen Memorial Hospital, Sun Yat-Sen University, 107 Yan Jiang West Road, Guangzhou, 510120 China; 3 Guangdong Provincial Key Laboratory of Malignant Tumor Epigenetics and Gene Regulation, Guangdong-Hong Kong Joint Laboratory for RNA Medicine, Sun Yat-Sen Memorial Hospital, Sun Yat-Sen University, Guangzhou, 510120 China; 4 Medical Research Center, Sun Yat-Sen Memorial Hospital, Sun Yat-Sen University, 107 Yan Jiang West Road, Guangzhou, 510120 China

**Keywords:** Colorectal cancer, mSEPT9, Prognosis

## Abstract

**Background:**

Abnormal hypermethylation of the septin 9 gene was an inchoate incident in some cancers. Though latest several researches had paid attention to its value in prognosis, the consequences were not distinctly, especially in colorectal cancer (CRC) with stage II and stage III.

**Purpose:**

The aim of this research was to pick up the prognostic value of the methylated septin 9 gene (mSEPT9) in CRC patients, particularly in TNM stage II—III.

**Methods:**

Blood samples before surgery were obtained from 144 CRC patients, of which there were 94 with stage II and stage III. mSEPT9 was considered positive when the cycle number of the peak reaction (Ct) was lower than the threshold value (41.0) for two times during three times PCR test. mSEPT9 and other relative factors of prognosis were estimated by survival analysis. The level of septin 9 in tissues was tested by immunohistochemical (IHC).

**Results:**

Stage II and stage III patients with mSEPT9 positive (mSEPT9+) had a lower disease-free survival (DFS) rate than those with mSEPT9 negative (mSEPT9-) (2-year DFS rates, 52.1% vs 73.9%, *P* = 0.014). In multivariate regression analysis, mSEPT9 was also an independent predictor of prognosis (*HR* = 2.741, *P* = 0.009). The risk of local recurrence or distant metastasis in CRC patients after surgery was mSEPT9+ with stage III, mSEPT9- with stage III/mSEPT9+ with stage II, and mSEPT9- with stage II (*P* = 0.001), from highest to lowest. In addition, mSEPT9 was strongly associated with TNM staging, tumor immersion depth, distant metastasis, differentiation degree, vascular invasion and microsatellite. When we explored the associations between septin 9 protein level revealed by IHC and other elements, recurrence/progression (*R* = − 0.523, *P* = 0.001), mSEPT9 status (*R* = − 0.451, *P* = 0.004) and T stage (*R* = − 0.375, *P* = 0.017) showed significant correlations.

**Conclusions:**

Positive mSEPT9 is a poor prognostic marker for CRC patients in stage II and III. It is also a powerful complement to TNM staging in predicting postoperative DFS of CRC patients of stage II and III.

## Introduction

CRC was one of the most prevalent tumors with high malignancy in the word, accounting for approximately 8% of the global cancer burden [1–3]. Surgical excision was the most important treatment for CRC, especially for patients with stage I-III disease [4]. Despite the thoroughness of initial radical resection, approximately 1/3 of patients would face CRC recurrence or metastatic death in stages II and III [5] and thus respond poorly to cancer treatment. TNM staging syetem, edited by American Joint Committee on Cancer was the principal guideline to predict patients prognosis in CRC [6]. But despite patients at the same stage, their outcomes were various, particularly in stage II and stage III [7]. Consequently, detecting high-risk patients with recurrence and metastasis in time and taking further intervention measures to improve the prognosis in stage II and III patients were very necessary. Some studies had suggested that the level of carcinoembryonic antigen before surgery was related to the prognosis of patients [8–10]. However, its sensitivity and specificity were not ideal [11]. Thus, more sensitive indicators should be identified and used as a supplementary part of TNM staging.

The septin 9 gene, located on chromosome 17, is responsible for encoding the septin 9 protein. The gene and its expression products are broadly involved in the growth and metabolic processes of the human body, including division, polarization, apoptosis, and so on [12]. Previous studies confirmed septin 9 gene and protein in CRC tissues played an anti-cancer effect in the generation and development of tumor. Nevertheless, CpG island hypermethylation of septin 9 promoter could suppress its normal expression, thereby inhibiting its effect [13]. Reports about mSEPT9 had been found in CRC, head and neck cancer, and gastric cancer [14, 15]. In addition, studies found that a tendency to distant metastasis could be observed in CRC patients with mSEPT9-positive in serum [14, 16, 17]. But the results of mSEPT9 still lacked unified standards in stage II and III.

This research aimed to pick up the prognostic value of mSEPT9 in CRC patients, particularly in TNM stage II and III. We hoped to achieve important prognostic outcomes at a low cost. Furthermore, we made a thorough inquiry into the relation between mSEPT9 and clinico-pathological features in CRC patients.

## Methods and materials

### Patients

**Fig. 1:**
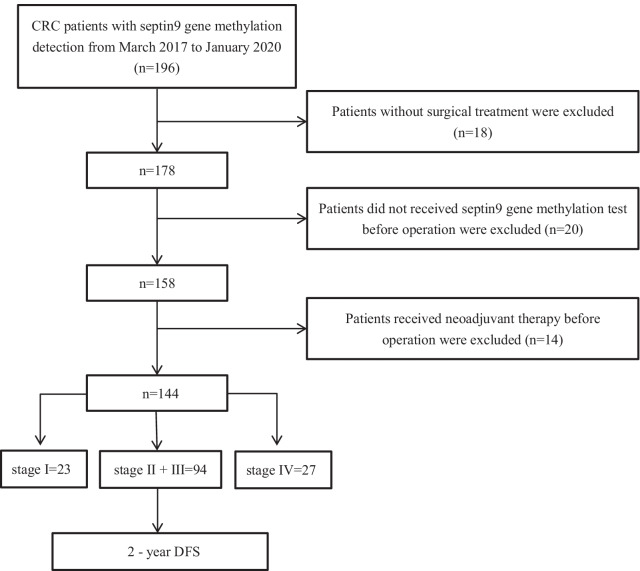
Flowchart of study design. Peripheral methylated septin 9 gene was detected preoperatively for clinico-pathological and prognosis analysis

To test the value of plasma mSEPT9, 144 of 196 patients with CRC at Sun Yat-sen Memorial Hospital, Guangzhou, China, between March 2017 and January 2020 were enrolled in this study. Following were the inclusion criteria: (I) primary CRC; (II) pathological diagnosis of CRC was determined according to World Health Organization criteria; (III) full clinical information available; (IV) patients received the detection of the methylation of septin 9 gene. The exclusion criteria were described below: (I) patiens did not received surgical treatment; (II) patients did not received septin 9 gene methylation test before surgery; (III) neoadjuvant treatment, including radiation or chemotherapy, was received; The specific inclusion and exclusion were shown in Fig. 1. Chemotherapy regimens for patients were based on NCCN Guidelines [18, 19]. In detail, for the most stage II and all stage III patients, CapeOx and mFOLFOX6 regimen are recommended in priority by the guidelines. As for the T3N0M0 patients in stage II, when there were no risk factors, clinical observation was adopted.

DFS was defined as the time between radical resection of the primary tumor until tumor recurrence or metastasis. Computed Tomography or Magnetic Resonance Imaging was used to monitor the recurrence or metastasis of tumor every 3–6 months. Patients had health checkups by phone every 3–6 months after chemotherapy. Postoperative review strategies were based on NCCN Guidelines [18, 19]. Each patient returned to the hospital every 3 months in the first year and every 6 months in the second and third year for the examination of chest, abdomen and pelvis after surgery or chemotherapy. The median follow-up time was 22.0 months. The final follow-up ended on May 31, 2021.

### Methylated septin 9 gene detection

The detection process of mSEPT9 was carried out in strict accordance with the instructions of the EPI Procolon 2.0 CE Kit (provided by Beijing Borcheng Technology Co., Ltd.), and the operation steps were as follows: (1) DNA extraction; (2) Sulfite transformation; (3) Bis DNA binding; (4) Bis DNA purification; (5) PCR of DNA samples using an ABI 7500 fastdx PCR instrument; And (6) using of the interpretation standard which was carried out according to the instructions of the kit. The human housekeeping gene β-actin was selected as the internal reference for PCR. PCR was performed three times. If the Ct was lower than the threshold value (41.0) two times, it was judged as positive; otherwise, it was negative.

### Immunohistochemistry

Septin 9 protein was detected in tumor tissues by IHC in 40 of 94 CRC samples in stage II and III. 3 fields (× 100, × 400) were randomly selected, and the staining results were determined according to the staining intensity of positive cells and the proportion of positive cells in the tissue. Results semi-quantitative accumulation method was used to determine the specific scoring criteria: (1) Dyeing degree: four different visual chromaticity were rated as 0 point, 1 point, 2 point and 3 point respectively for non-staining, light yellow, brown-yellow and tan. (2) Number of positive cells: 0–5% cells was 0 point, 6–25% was 1 point, 25–50% was 2 point, > 50% was 3 point. The best cutoff value of the score was decided using the receiver operation characteristic (ROC) curve (Fig. 5a). When the two items were added together, a total score < 3 was regarded as low expression, while a total score >  = 3 was regarded as high expression.

### Statistical analysis

SPSS 25.0, GraphPad Prism 8, and R 3.6.1 were applied to statistical analyses and graphics. Chi-square tests and Fisher’s exact tests were devoted to analyzing the connection between mSEPT9 and other clinico-pathological elements. K-M log-rank test and univariate analysis were used to investigate the relationship between mSEPT9 and DFS, and the variables with a *P* < 0.05 were adopted into the multivariate Cox regression analysis. The correlation between the expression of septin 9 protein in tumor tissue and cancer progression was evaluated using Spearman rank correlation analysis. Based on the results of multivariate analysis, a nomogram of recurrence or metastasis probability was formed. A *P* < 0.05 for 2-side was considered statistically significant.

## Results

### Serum mSEPT9-positive was closely related to the advanced-stage of CRC

**Fig. 2:**
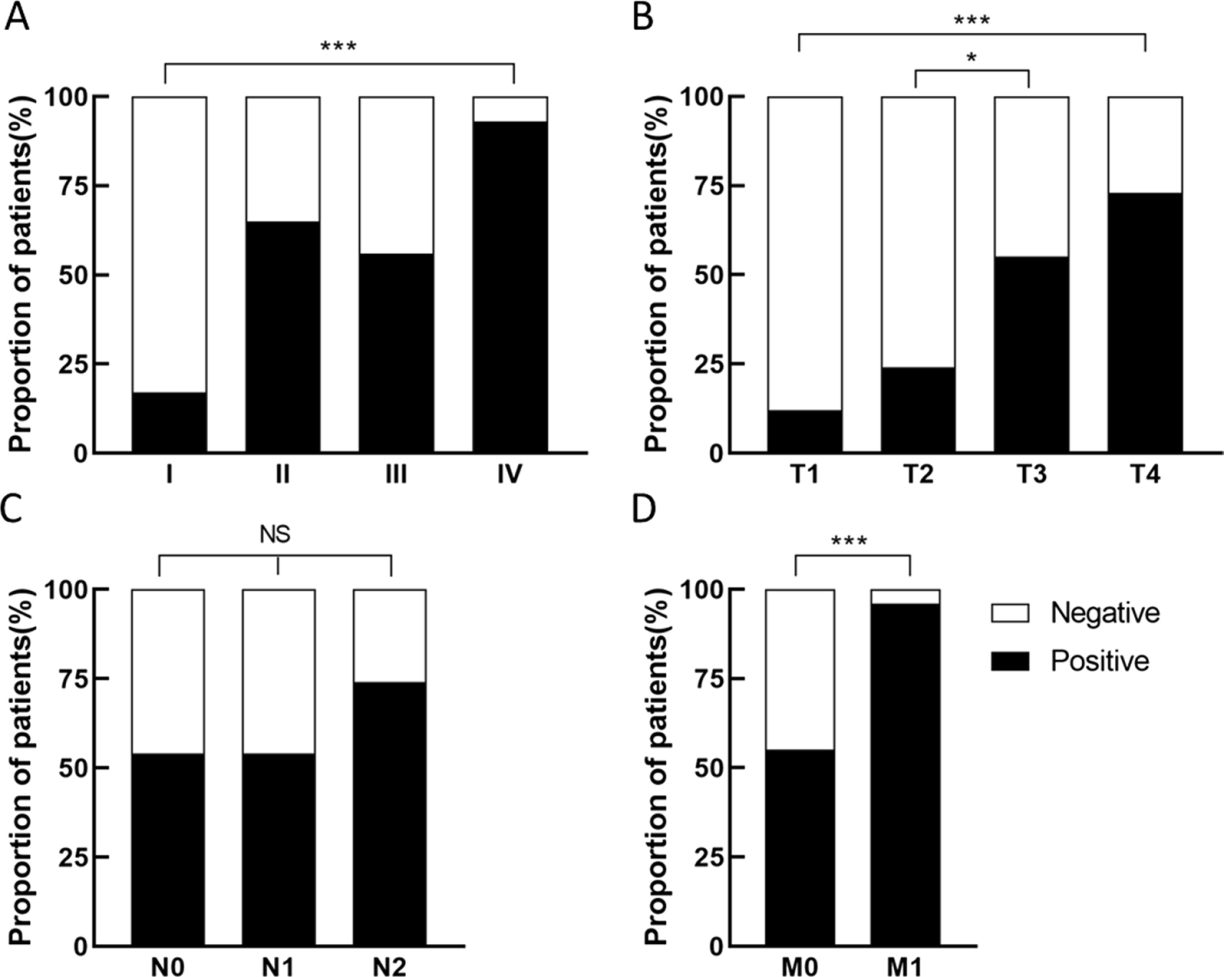
The proportion of septin 9 gene methylation positive and negative patients under different states. **a** TNM stage; **b** T stage; **c** N stage; **d** M stage. *P < 0.05; ***P < 0.001; NS: no signification

Patients' complete baseline features and mSEPT9 groups of negative and positive were shown in Table 1. In the study, vascular invasion (*P* = 0.020), microsatellites (*P* < 0.001), histologic grade (*P* = 0.006), TNM stage (*P* < 0.001), tumor infiltration depth (*P* < 0.001) and distant metastasis (*P* < 0.001) were significantly associated with mSEPT9. No significant connection could be seen in gender, age, tumor location, and lymphatic metastasis with mSEPT9. Notably, we further explored the correlation between the status of mSEPT9 and TNM stage. The rate of mSEPT9+ in IV was significantly higher than that in stage I-III, especially in I (92.6% vs 17.4%, *P* < 0.001, Fig. 2a). We also analyzed the association of the rate of positive mSEPT9 with the T stage (T1–T4), N stage (N0–N2), M stage (M0–M1), alonely. The rate of mSEPT9+ revealed a significant increase from T1 to T4 (Fig. 2b), and mSEPT9+ rate in T4 was higher than that in T1 (72.2% vs 12.5%, *P* < 0.001). N stage with a slight change in the rate of mSEPT9+ and did not show connection (Fig. 2c). As shown in Fig. 2d, mSEPT9 had an excellent ability to make a distinction between local and metastatic CRC (*P* < 0.001).

**Table 1:** Correlations between serum methylated septin 9 gene and clinico-pathological features of colorectal cancer patients (n = 144) (Chi square and Fisher’s tests)

Parameter	Negative group (%)	Positive group (%)	*P*
Gender			
Male	31(36.0)	55(64.0)	0.121
Female	29(50.0)	29(50.0)	
Age			
≦60 yr	26(41.9)	36(58.1)	0.955
>60 yr	34(41.5)	48(58.5)	
T stage			
T1	7(87.5)	1(12.5)	< 0.001
T2	13(76.5)	4(23.5)	
T3	18(45.0)	22(55.0)	
T4	22(27.8)	57(72.2)	
N stage			
N0	38(46.3)	44(53.7)	0.116
N1	13(46.4)	15(53.6)	
N2	9(26.5)	25(73.5)	
Distant metastasis (M)			
M0	58(49.6)	59(50.4)	< 0.001
M1	2(7.4)	25(92.6)	
TNM stage			
I	19(82.6)	4(17.4)	< 0.001
II	19(38.8)	30(61.2)	
III	20(44.4)	25(55.6)	
IV	2(7.4)	25(92.6)	
Vascular invasion			
Absent	51(47.7)	56(52.3)	0.020
Present	9(24.3)	28(75.7)	
Histologic grade			
Low level	1(7.1)	13(92.9)	0.006
Medium level	57(44.9)	70(55.1)	
High level	2(66.7)	1(33.3)	
Location			
Right Colon	10(30.3)	23(69.7)	0.064
Left Colon	16(34.8)	30(65.2)	
Rectum	34(52.3)	31(47.7)	
Microsatellite			
pMMR	59(45.0)	72(55.0)	0.008
dMMR	1(7.7)	12(92.3)	

pMMR: proficient mismatch repair; dMMR: deficient mismatch repair

### mSEPT9 was an independent risk factor for local recurrence or distant metastasis in stage II and stage III CRC patients after surgery.

At the follow-up period, 38.3% (36/94) of patients underwent local recurrence or distant progression, with 12 of stage II and 24 of stage III. The DFS rates were 73.4% and 61.4% at 12 and 24 months, respectively. The univariate analysis was revealed in Table 2. mSEPT9, TNM stage, tumor infiltration depth and lymphatic metastasis were significant with DFS (*P* < 0.05).

**Table 2:** Univariate and multivariate analysis of disease free survival in CRC patients with stage II-III

Parameter	Univariate analysis, *HR* (95% CI)	*P*	Mutivariate analysis, *HR* (95% CI)	*P*
Gender				
Male	1 (Referent)	0.384	–	–
Femal	0.728 (0.357–1.488)	–	–	–
Age				
≦60 yr	1 (Referent)	0.929	–	–
>60 yr	0.970 (0.499–1.887)	–	–	–
T stage				
T2-3	1 (Referent)	0.007	–	–
T4	3.346 (1.388–8.065)	–	–	–
N stage				
N0	1 (Referent)	< 0.001	–	–
N1	1.312 (0.508–3.384)	–	–	–
N2	5.290 (2.464–11.355)	–	–	–
TNM stage				
II	1 (Referent)	0.005	1 (Referent)	0.003
III	2.789 (1.365–5.700)	–	3.010 (1.470–6.163)	
Vascular invasion				
Absent	1 (Referent)	0.085	–	–
Present	2.006 (1.015–3.964)	–	–	–
Location				
Right Colon	1 (Referent)	0.090	–	–
Left Colon	0.345 (0.128–0.933)			
Rectum	0.858 (0.405–1.817)	–	–	–
Microsatellite				
pMMR	1 (Referent)	0.118	–	–
dMMR	2.129 (0.825–5.494)	–	–	–
mSEPT9				
Negative	1 (Referent)	0.020	1 (Referent)	0.009
Positive	2.509 (1.175–5.359)	–	2.741 (1.281–5.865)	–

pMMR: proficient mismatch repair; dMMR: different mismatch repair;

mSEPT9: methylated septin 9 gene; CRC: colorectal cancer

mSEPT9 showed a strong relation with DFS. Stage II and stage III patients with mSEPT9+ had a lower DFS rate than those with mSEPT9- (2-year DFS rates, 52.1% vs 73.9%, *P* = 0.014, Fig. 4d). The multivariate analysis informed that mSEPT9 had independent prognostic significance for CRC patients (*HR* = 2.741, *P* = 0.009), as well as TNM stage (*HR* = 3.010, *P* = 0.006) (Table 2).

**Fig. 3:**
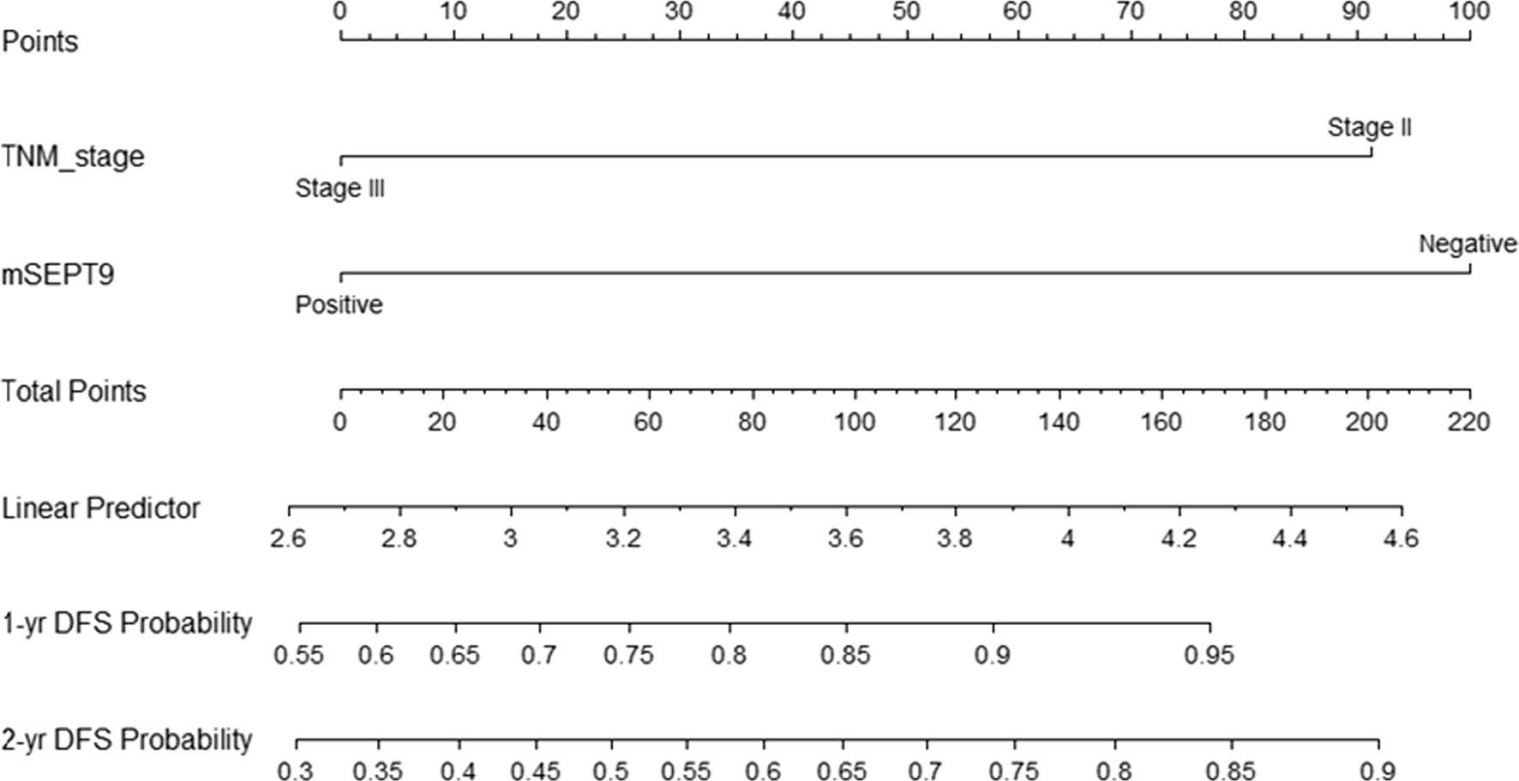
A nomogram to predict the risk of local recurrence and distant metastasis for colorectal cancer patients

According to above analysis, mSEPT9 and TNM stage were selected to found the nomogram to appraise the risk of relapse or progression of CRC patients (Fig. 3). Each factor corresponds to a number on the scale axis. By adding each score, clinicians and patients could easily calculate the 1-year and 2-year probabilities of DFS.

### Combining mSEPT9 with TNM stage could better judge the prognosis of CRC patients.

**Fig. 4:**
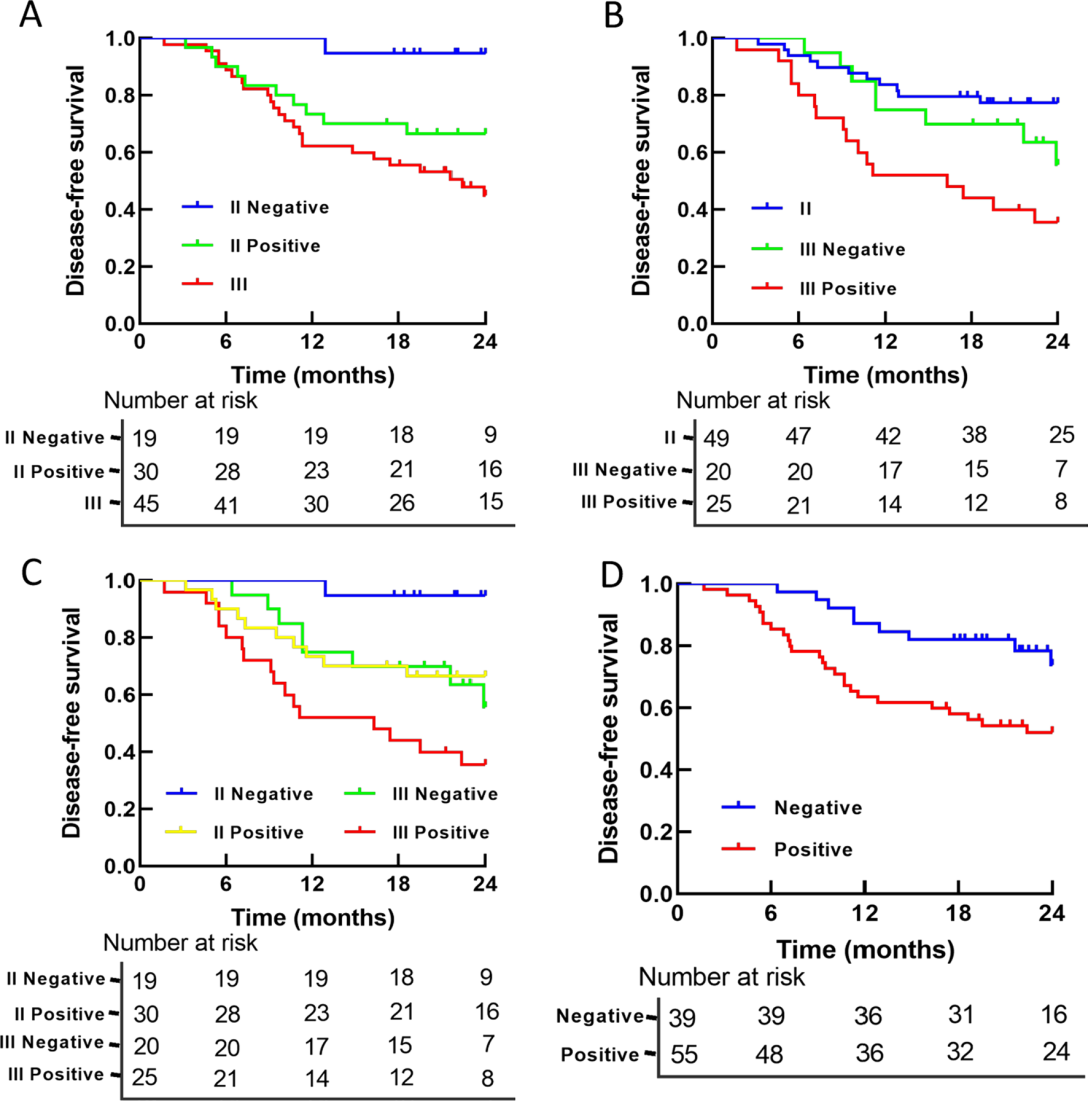
Survival curves based on TNM stage and mSEPT9. **a–c** TNM stage and mSEPT9 were combined for DFS analysis. **d** DFS was analyzed according to mSEPT9 status. DFS: disease free survival; mSEPT9: methylated septin 9 gene

Based on mSEPT9, the survival was further analyzed. In terms of 2-year DFS, patients with stage II mSEPT9+ performed significantly worse than those with stage II mSEPT9- (*P* = 0.023, Fig. 4a), but no statistical difference could be seen between mSEPT9+ and mSEPT9- patients in stage III (*P* = 0.078, Fig. 4b). Besides, although patients with stage II mSEPT9- had better survival than patients in stage III (*P* = 0.001, Fig. 4a), patients with mSEPT9+ II had similar survival as patients in stage III (*P* = 0.132, Fig. 4a). In the meantime, the survival rate between mSEPT9- in stage III and patients of stage II was close (*P* = 0.183, Fig. 4b). Survival was significantly lower in stage III mSEPT9+ patients than in stage II patients (*P* < 0.001, Fig. 4b).

DFS was further stratified by TNM staging and mSEPT9, all results were shown in Fig. 4c. mSEPT9- stage II patients had better DFS than mSEPT9- patients of stage III (*P* = 0.012). On the survival curve, the stage II mSEPT9+ patients and stage III mSEPT9- patients almost overlapped (*P* = 0.761). As for mSEPT9+ patients in stage II and III, a significant difference could also be seen (*P* = 0.032). Overall, patients had the risk of recurrence or metastasis in the sequence of stage III mSEPT9+, stage III mSEPT9-/stage II mSEPT9+ , and stage II mSEPT9- (*P* = 0.001), from high to low.

### Low expression of septin 9 protein in tumor tissues was often related to poor prognosis.

**Fig. 5:**
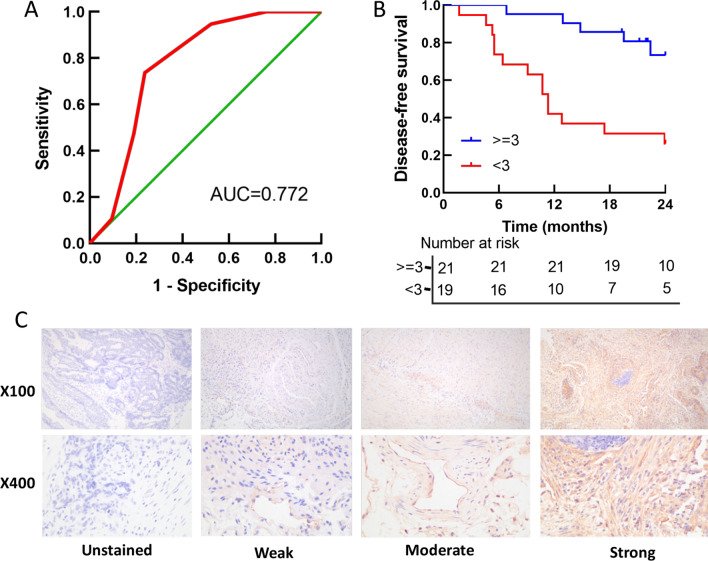
**a** Receiver operating characteristic curve to determine the optimal cutoff value for septin 9 protein expression scores. **b** Survival analysis of septin 9 protein expression with prognosis. **c** Immunohistochemistry assay of septin9 protein expression in colorectal cancer tissues

To investigate the clinical relevance between the septin 9 expression in tumor tissue and cancer progression, septin 9 protein was detected by IHC in 40 CRC patients, including 20 cases in stage II and III, separately (Table 3). The cytoplasmic region with brownish-yellow particles was positively expressed (Fig. 5c). Of these tumors, 21 showed high expression, while the remaining 19 showed no or little detectable staining. Patients with low expression of septin 9 in tumor tissue showed significantly poor DFS (*P* < 0.001, Fig. 5b). The connection between septin 9 expression and important prognostic risk factors was verified by Spearman correlation analysis, which included recurrence/progression (*R* = − 0.523, *P* = 0.002), mSEPT9 status (*R* = − 0.451, *P* = 0.004), and T stage(*R* = − 0.375, *P* = 0.017) (Table 4).

**Table 3:** Clinico-pathological features and septin 9 protein expression in colorectal cancer tissue (n = 40)

Parameter	No. of cases (%)
Age	
≦60 yr	15 (37.5)
>60 yr	25 (62.5)
Gender	
Male	26 (65.0)
Female	14 (35.0)
Location	
Right colon	7 (17.5)
Left colon	15 (37.5)
Rectum	18 (45.0)
T stage	
T2	2 (5.0)
T3	12 (30.0)
T4	26 (65.0)
N stage	
N0	20 (50.0)
N1	7 (17.5)
N2	13 (32.5)
TNM stage	
II	20 (50.0)
III	20 (50.0)
Septin 9 of tissue	
Strong	21 (52.5)
Weak	19 (47.5)
mSEPT9	
Positive	23 (57.5)
Negative	17 (42.5)
Recurrence/progression	
Occurrence	19 (47.5)
Nothingness	21 (52.5)

mSEPT9: methylated septin 9 gene

**Table 4:** The correlation between the expression of septin 9 protein in tissues and clinico-pathological features (n = 40) (Spearman analysis)

Parameter	The expression of septin 9 protein	
	Spearman correlation	*P *value
T stage	− 0.375	*P* = 0.017
N stage	− 0.258	*P* = 0.108
Vascular invasion	− 0.173	*P* = 0.286
mSEPT9 status	− 0.451	*P* = 0.004
Recurrence/progression	− 0.479	*P* = 0.002

mSEPT9: methylated septin 9 gene

## Discussion

Although TNM staging is the most important tool to judge the prognosis of colorectal cancer after surgery, some restrictions remained. For instance, patients might have different outcomes regardless of the same stage, especially in stage II and stage III patients [7]. More and more attention has been received in mSEPT9 of CRC, but the results still lacked unified standards in stage II and stage III. In our study, 94 CRC patients in stage II and III were analyzed by combining mSEPT9. 38.3% of patients underwent local recurrence or distant metastasis at the 2-year follow-up, consistent with the previous report [23]. Our results confirmed that mSEPT9 was an independent prognosis marker for CRC patients in stage II and III. mSEPT9+ patients showed lower postoperative DFS than mSEPT9- patients. In addition to the above results, we also found that the mSEPT9+ in serum was closely related to the advanced-stage of CRC. Previous studies suggested that septin 9 protein played a tumor suppressive role in CRC [20, 21]. However, as the tumor progressed, more and more CpG islands of the septin 9 gene promoter were methylated, affecting its normal gene expression process [13]. As a result, the antitumor effect of the septin 9 protein was limited, leading to a poor prognosis. Our results further confirmed the above conclusion. Through the detection of spetin 9 protein in tumor tissues, we found that when septin 9 protein was low expressed in tumor tissues, its gene methylation level tended to be high in plasma, and low expression of septin 9 protein in tumor tissues was often associated with poor prognosis. Combined with the above results, we had reason to infer that methylated septin 9 gene in serum was an important factor causing the poor prognosis of patients.

And that, the consequences confirmed mSEPT9 was a powerful complement to TNM system. Further survival analysis proved that the survival rate of patients at stage II mSEPT9+ was comparable to that of patients in stage III, and that of patients in stage III mSEPT9- was equivalent to that of patients in stage II. Overlap survival curves for stage II mSEPT9+ and stage III mSEPT9- were observed. So the risk of local recurrence and distant metastasis for CRC patients after surgery could be stratified into three layers: (1) stage III mSEPT9+ ; (2) stage III mSEPT9- /stage II mSEPT9+ ; and (3) stage II mSEPT9-. Based on mSEPT9, stage II and III patients could be further classified to provide a basis for individualized adjuvant therapy. Previous studies only simply found that the status of mSEPT9 before surgery was associated with survival of CRC patients [22, 23], lacked in-depth understanding of its specific role. We stratified stage II and III patients in detail using mSEPT9 and clearly demonstrated the role of mSEPT9 in survival outcomes for the first time. Approximately 30% to 50% of patients with stage III CRC were at risk for recurrence or metastasis despite thorough excision [24]. And for stage II patients, the 2-year DFS rate was approximately 60% to 80% [25]. Chemotherapy could increase the survival rate of CRC patients [26–28]. Finding high-risk patients as early as possible and giving them individualized intervention could greatly improve the survival rate of patients [29].

Our study also revealed some interesting associations between mSEPT9 and clinicopathological factors. In our research, mSEPT9 performed well as an adjunct molecular staging parameter. A high rate of mSEPT9+ was connected with advanced tumor infiltration and metastasis, but not with lymphatic metastasis. These results add valuable information to the classification of tumors [30, 31], and help to guide clinicians to improve examination and treatment plans. In addation, when patients were with deficient mismatch repair, the positive rate of mSEPT9 was at a high level, these results suggested that mismatched repair proteins may play an important role in the methylation of septin 9 gene.

In our research, there are also some limitations. Firstly, in our study, the number of stage II and III patients was relatively small, and the conclusion of further study should be supported by more data. Secondly, due to the short follow-up time, the long-term survival of patients could not be further explored. Finally, our conclusions are not further verified by external data. In the future, multi-center studies on the relationship between septin 9 gene methylation and prognosis in CRC patients should be carried out.

## Conclusion

In conclusion, positive mSEPT9 is a poor prognostic marker for stage II and III CRC patients. It is also a powerful complement to TNM staging in predicting postoperative DFS of CRC patients of stage II and III.

## Data Availability

Data supporting the results of this study are available from the corresponding author upon reasonable request.

## Abbreviations

CRC: Colorectal cancer
CI: Confidence interval
HR: Hazard ratio
mSEPT9: Methylated septin 9 gene
Ct: Cycle number of the peak reaction
IHC: Immunohistochemical
DFS: Disease-free survival
mSEPT9+: mSEPT9 positive
mSEPT9-: mSEPT9 negative
ROC: Receiver operation characteristic
AUC: Area under the ROC curve
pMMR: Proficient mismatch repair
dMMR: Deficient mismatch repair
